# Geometric Complexity Control in Topology Optimization of 3D-Printed Fiber Composites for Performance Enhancement

**DOI:** 10.3390/ma17092005

**Published:** 2024-04-25

**Authors:** Tao Wu, Peiqing Liu, Jikai Liu

**Affiliations:** Key Laboratory of High Efficiency and Clean Mechanical Manufacture (Ministry of Education), School of Mechanical Engineering, Shandong University, Jinan 250061, China; wu_tao@mail.sdu.edu.cn (T.W.); lpq17@mail.sdu.edu.cn (P.L.)

**Keywords:** topology optimization, fiber composites, 3D printing, geometric complexity

## Abstract

This paper investigates the impact of varying the part geometric complexity and 3D printing process setup on the resulting structural load bearing capacity of fiber composites. Three levels of geometric complexity are developed through 2.5D topology optimization, 3D topology optimization, and 3D topology optimization with directional material removal. The 3D topology optimization is performed with the SIMP method and accelerated by high-performance computing. The directional material removal is realized by incorporating the advection-diffusion partial differential equation-based filter to prevent interior void or undercut in certain directions. A set of 3D printing and mechanical performance tests are performed. It is interestingly found that, the printing direction affects significantly on the result performance and if subject to the uni direction, the load-bearing capacity increases from the 2.5D samples to the 3D samples with the increased complexity, but the load-bearing capacity further increases for the 3D simplified samples due to directional material removal. Hence, it is concluded that a restricted structural complexity is suitable for topology optimization of 3D-printed fiber composites, since large area cross-sections give more degrees of design freedom to the fiber path layout and also makes the inter-layer bond of the filaments firmer.

## 1. Introduction

Manufacturability has been a long lasting issue for topology optimization [1]. The geometric complexity, sourced from pursuing extreme mechanical performance, makes the manufacturing non-trivial, e.g., frequently encountering machining tool interference [2,3,4] or demolding obstacles by enclosed voids/undercuts [5,6]. Hence, manufacturing oriented topology optimization has been highly focused during the past three decades. The emergence of additive manufacturing technology has to some extent relieved the manufacturability issue. The bottom-up slicing-based material deposition enables freeform manufacturing. Sacrificial supports are added to mechanically back up the manufacturing process [7]. Hence, the topological designs, regardless of complexity, are friendly to additive manufacturing. On the other hand, research efforts were put into the topology optimization algorithm development dedicated to additive manufacturing, attempting to reduce the expense or improve the quality of combining these two techniques [8]. For instance, self-support topology optimization aims at eliminating the need for support since they consume extra materials and printing time, and removing the support is tedious. Both the 45-degree inclination rule [9,10] and the minimum overhang size rule have been developed to impose the self-support restriction. In situ strength constraints are developed for topology optimization to prevent in-process part failure due to insufficient layer-wise bonding. This manufacturability restriction applies to both the Stereolithography process for photosensitive resins [11] and the Selective Laser Melting for metals [12,13]. For the Directed Energy Deposition metal 3D printing, the poor shape and surface quality requires post-process subtractive machining for amendment, and hence, topology optimization for hybrid manufacturing emerges as a hot topic [14] that incorporates multi-axis accessibility and additive–subtractive process planning [15,16] as essential elements.

Regarding fiber-composite 3D printing [12], it is majorly conducted based on the material extrusion process, e.g., the Fused Deposition Modeling (FDM). There are tremendous process features that affect the printing performance [13,17], e.g., build direction, path pattern, layer height, printing speed, extrusion rate, etc. These factors have been extensively investigated through experiments. Regarding design, the most closely linked factors are build direction and path pattern, since material extrusion type 3D printing exhibits evident property anisotropy and the fiber-reinforcement enhances the anisotropy. Hence, topology optimization for fiber composites should include the direction-related information, making the concurrent optimization effect. Liu and Yu [18] developed the early method that the level set function concurrently models the structural geometry and the contour-offset deposition paths, and the level set gradient facilitates evaluating the local path directions and thus builds the orthotropic constitutive law. Hence, the Hamilton–Jacobi equation-based boundary evolution [19] concurrently updates the structure and its infilling deposition paths. This optimization framework was followed up in a few later publications, addressing the contour-offset and zigzag hybrid path pattern [20] and even the skeleton-based path pattern [21]. The challenging domain-to-boundary sensitivity transformation was addressed in [22]. Another concurrent optimization strategy is to decompose the part into subdivisions and then, simultaneously optimize the subdivision shapes and also the infill path patterns [23]. For instance, the DMO (discrete material modeling) [24] can be applied to interpolate the multiple subdivisions [25], and the path-wise deposition paths can be just linear or a distance field. At the end, we would mention a more general optimization idea by treating the fiber angels as local direction variables [26]. The gradient information can efficiently optimize the fiber orientation field, but the thereby obtained fiber directions would be chaotic as the path directions from neighboring elements can be totally differently [27]. Then, transforming the discrete directions into an effective continuous fiber deposition path would be troublesome. The streamline method can be applied to extract continuous fiber paths [28] but it severely deviates some optimized local directions and the streamlines would not be equidistant. Adding smoothing filters to direction variables during optimization [29] has been attempted, which alleviates the issue but cannot eliminate it. Recently, neural networks were also applied to realize topology optimization for fiber composites [30].

Given the reported works that incorporate both topology optimization and fiber composite 3D printing practice, topology optimization of continuous fiber reinforced composite (CFRC) structures is more challenging because CFRC 3D printing has a strict constraint on the continuity of printing paths. Chen and Ye [31] calculated the average load transmission trajectories of the structure to defined the continuous carbon fiber placement paths for CFRC 3D printing. There is a significant reinforcement effect of short carbon fiber and continuous carbon fiber on specimens printed with pure polyamide (PA). Yang et al. [32] proposed an orthogonal-element solid orthotropic material with a penalization (SOMP) topology optimization method for CFRC 3D printing. The experimental results showed the proposed method realized increments in stiffness by 30%. Jiang et al. [26] enhances the traditional SIMP approach by including fiber angle as a design variable for the optimization of CFRC structures. The effectiveness of the method was validated by three-point bending tests of MBB beam.

The existing topology optimization results for fiber composites are mainly 2.5D structures. This leaves an open question for the same design, would the 3D freeform topological design perform beyond the 2.5D extruded structure if testing the print-outs? The 3D topology optimization result may have many structural details; however, when slicing the printing layers, these details may transform into isolated small areas that fall short in shaping and bonding quality. Then, would a geometrically simplified 3D design beat the freeform 3D design from a real test? Hence, we would work on topology optimization for fiber composites subject to different schemes: 2.5D topology optimization, 3D freeform topology optimization, and 3D simplified topology optimization with directional material removal. Their performances would be evaluated based on real tests and hopefully, the experimental observations would benefit the community for configuring the appropriate topology optimization algorithm to design for the best fiber-reinforcement effect.

## 2. Topology Optimization Method

### 2.1. Topology Optimization for 2.5D Fiber Composite Structures

The 2.5D structure is obtained by extruding the 3D topological design through a certain thickness which, therefore, would have the identical cross-section. According to the finite element method, the stiffness matrix of a two-dimensional solid element for linear elasticity can be calculated by Equation (1):

(1) $$k_{0}=\int_{-1}^{+1}\int_{-1}^{+1}B^{T}D_{0}B{d\xi }_{1}{d\xi }_{2}$$

where $D_{0}$ is the constitutive matrix with unit Young’s modulus, $\xi_{i}$ represents the natural coordinate system, and B is the strain-displacement matrix. The global stiffness matrix K can be assembled by the following:

(2) $$K=\overset{N}{\underset{e=1}{\sum }}k_{e}\left(\rho_{e}\right)=\overset{N}{\underset{e=1}{\sum }}E\left(\rho_{e}\right)k_{0}$$

where N is the total number of elements. The Young’s modulus $E\left(\rho_{e}\right)$ is interpolated by the SIMP (Solid Isotropic Material with Penalization) approach [33],

(3) $$E\left(\rho_{e}\right)=E_{\min}+\rho_{e}^{p}\left(E_{0}-E_{\min}\right)$$

where $\rho_{e}$ represents the element density that varies continuously between 0 and 1. $E_{0}$ is the Young’s modulus of the material, and $E_{\min}$ is set to 1 × 10^−5^ for voids to prevent the stiffness matrix singularity. p is a penalty factor introduced to derive the close to black-and-white density field solution, usually given the value of 3. Finally, the nodal displacements can be obtained by solving the finite element equilibrium Equation (4),

(4) KU=F

where **U** and **F** are the global displacement and force vectors, respectively.

The mathematical formulation of the optimization problem for structural compliance minimization is shown as follows,

(5) $$\begin{array}{c} \underset{\rho }{\min}\;c\left(\rho \right)=U^{T}KU=\overset{N}{\underset{e=1}{\sum }}E\left(\rho_{e}\right)u_{e}^{T}k_{0}u_{e}\\ s.t.\;\;\;\;\;\;\;\;KU=F\\ \;\;\;\;\;\;\;\;\;\;\;\;\;\;V\left(\rho \right)/V_{0}\leq fv\\ \;\;\;\;\;\;\;\;\;\;\;\;\;\;0\leq \rho \leq 1 \end{array}$$

where c is the compliance,$\;u_{e}$ is the element displacement vector. V(ρ) and $V_{0}$ are the volumes of the solid domain and the total design domain, respectively. fv is the predefined volume fraction limit.

In order to ensure the existence of solutions and to avoid the checkerboard structure, some restrictions are applied on the design variables. This work adopts the Helmholtz type PDE filter [34] for smoothing,

(6) $$-r^{2}\nabla^{2}\bar{\rho }+\bar{\rho }=\rho$$

where $\bar{\rho }$ represents the filtered density field, r is the length parameter. The minimum length scale of the structure is associated with the length parameter.

Then, the grey boundary issue is solved by applying the Heaviside projection [35]. The approximated Heaviside function is given by,

(7) $$\tilde{\rho }=\frac{\tanh\left(\beta \eta \right)+\tan h(\beta (\bar{\rho }-\eta ))}{\tanh\left(\beta \eta \right)+\tanh\left(\beta \left(1-\eta \right)\right)}$$

where η∈(0, 1) is the threshold. When the value of $\bar{\rho }$ is greater than this threshold, $\tilde{\rho }$ tends to be 1, otherwise it tends to be 0. β controls the steepness of the projection function. When β→∞, the value of $\tilde{\rho }$ is exactly 0 and 1; however, the function is no longer smooth, which impedes a stable convergence. Therefore, it is suggested to adopt a small β at the beginning and gradually increase it to the upper bound.

The sensitivity of the objective function c with respect to the design variable ρ can be derived by the chain rule,

(8) $$\frac{\partial c}{\partial \rho_{e}}=\frac{\partial c}{\partial {\tilde{\rho }}_{e}}\frac{\partial {\tilde{\rho }}_{e}}{\partial {\bar{\rho }}_{e}}\frac{\partial {\bar{\rho }}_{e}}{\partial \rho_{e}}$$

where $\frac{\partial {\tilde{\rho }}_{e}}{\partial {\bar{\rho }}_{e}}$ and $\frac{\partial {\bar{\rho }}_{e}}{\partial \rho_{e}}$ modifies the sensitivity according to the Heaviside filter and PDE filter, respectively. $\frac{\partial c}{\partial {\tilde{\rho }}_{e}}$ can be derived from Equations (3) and (5).

(9) $$\frac{\partial c}{\partial {\tilde{\rho }}_{e}}=-p{\left({\tilde{\rho }}_{e}\right)}^{p-1}\left(E_{0}-E_{\min}\right)u_{e}^{T}k_{0}u_{e}$$

$\frac{\partial {\tilde{\rho }}_{e}}{\partial {\bar{\rho }}_{e}}$ can be obtained from Equation (7), and $\frac{\partial \bar{\rho }\;}{\partial \rho }$ can be expressed as follows:

(10) $$\frac{\partial {\bar{\rho }}_{\;}}{\partial \rho_{\;}}=T^{T}K_{F}^{-1}$$

where T is a transformation matrix which maps the element density vector to the node density vector, and $K_{F}^{\;}$ is the standard FE stiffness matrix for solving Equation (6). More details of the derivation can be found in [34,36].

### 2.2. Topology Optimization for 3D Fiber Composite Structures

The 3D topology optimization problem is extended from the 2D version by adding another dimension to the formulas in Section 2.1. Hence, the algorithm details tend to be similar and thus, would not be repeated.

**Figure 1:**
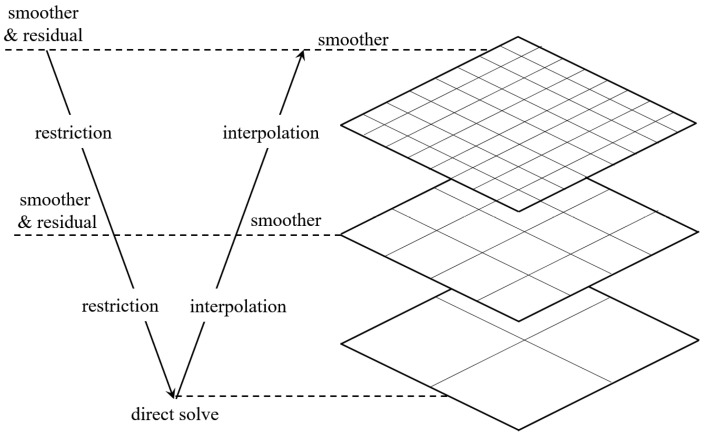
Schematic diagram of the multigrid method in V-Cycle mode.

On the other hand, the degrees of freedom in a 3D problem extend significantly compared with the 2D version. The scale of global stiffness matrix **K** can be too large due to the limited computer memory and computing resource and solving Equation (4) can be computationally prohibitive. Therefore, a high-performance paralleled solver based on OpenMP [37] is developed. The preconditioned conjugate gradient method is used to iteratively solve the large-scale linear equations. The solver also adopts the multigrid technology in the V-Cycle mode to obtain a fast solution on the sparse grid and compensate the error based on the fine grid analysis. The schematic diagram of the multigrid method is summarized in Figure 1. In each V-Cycle, the grid is smoothed, and the residual is calculated and propagated to the coarser grid. At the coarsest level, a direct solver is applied, and then the solution is iteratively interpolated into the finer grids.

### 2.3. 3D Topology Optimization Algorithm with Directional Material Removal

The optimized 3D structure may have many branch features, which predictably increases the deformation resistance of the structure. However, for fiber composite 3D printing, the many structural branches would create tremendous, small-area cross-sections that are non-preferred by filament extrusion type 3D printing, since both the inter- and intra-layer bonding quality would be compromised. Length scale control [38,39] can be applied to restrict the cross-section sizes, but its adoption for large structural changes may make the algorithm unstable. Hence, we propose directionally adding and removing materials during topology optimization, controlling the structural complexity and preventing isolated islands during slicing.

Specifically, an advection-diffusion PDE-based filter is adopted that originally models the light projection. The filtering equation is expressed as follows [40],

(11) $$\begin{array}{c} -L^{2}\nabla \cdot \left(A\nabla \psi \right)+Lv\cdot \nabla \psi =\beta \tilde{\rho }\left(1-\psi \right)\\ \{\begin{array}{c} \psi =0,\;\;\mathrm{on}\Gamma_{D}:\Gamma_{D}\subset \Gamma ,n\cdot v\leq 0\\ \nabla \psi \cdot n=0,\;\;\mathrm{on}\;\Gamma_{N},\;\left\{\Gamma_{N}:\Gamma \backslash \Gamma_{D}\right\} \end{array} \end{array}$$

where ψ represents the filtered density field, L is the length along the convection direction of the design domain, A is the diffusion matrix, and v is the advection vector that aligns with the material change direction (the value of ‖v‖ is usually 1000). β is the source magnitude term, and Γ is the boundary of the design domain. $\Gamma_{D}$ and $\Gamma_{N}$ represent the Dirichlet boundary and Newman boundary, respectively, and n is the boundary normal vector. The specifications of v, L and A are given in Equations (12)–(14).

(12) $$\begin{array}{c} v=\|v\|\left[\sin\left(\varphi \right)\cos\left(\theta \right),\cos\left(\varphi \right),\;\sin\left(\varphi \right)\sin\left(\theta \right)\right]\\ \varphi \in \left[0,\;\pi \right],\;\theta \in \left[0,\;2\pi \right] \end{array}$$

**Figure 2:**
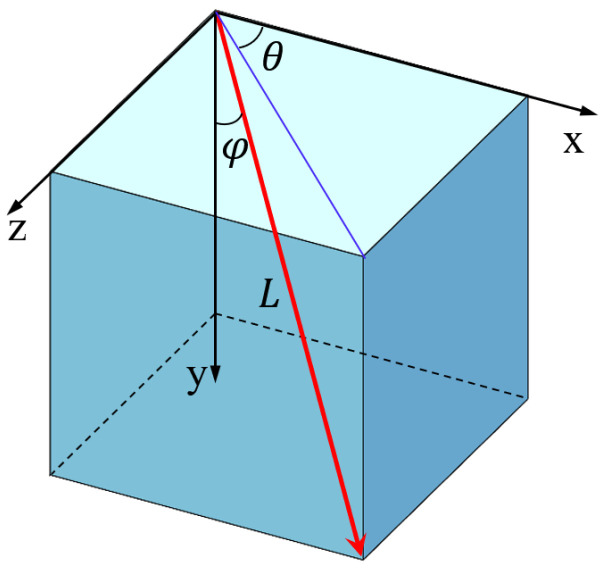
Schematic diagram of coordinate transformation angles.

θ and φ are the including angles of the material change direction relative to the axes x and y, respectively, as shown in Figure 2.

(13) $$L=L_{x}{\left({\left(\sin\left(\varphi \right)\cos\left(\theta \right)\right)}^{2}\right)}^{\frac{1}{2}}+L_{y}{\left({\left(\cos\left(\varphi \right)\right)}^{2}\right)}^{\frac{1}{2}}+L_{z}{\left({\left(\sin\left(\varphi \right)\sin\left(\theta \right)\right)}^{2}\right)}^{\frac{1}{2}}$$

$L_{x}$, $L_{y}$ and $L_{z}$ are the lengths of the design domain in the x, y and z directions, respectively.

(14) $$A=10^{-2}+\left[\begin{array}{ccc} {\left({\left(\sin\left(\varphi \right)\cos\left(\theta \right)\right)}^{2}\right)}^{\frac{1}{2}} & 0 & 0\\ 0 & {\left({\left(\cos\left(\varphi \right)\right)}^{2}\right)}^{\frac{1}{2}} & 0\\ 0 & 0 & {\left({\left(\sin\left(\varphi \right)\sin\left(\theta \right)\right)}^{2}\right)}^{\frac{1}{2}} \end{array}\right]$$

**Figure 3:**
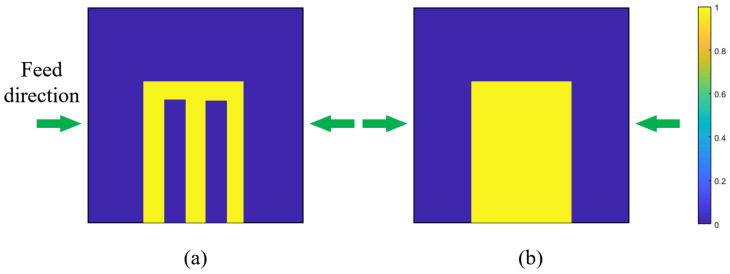
Schematic diagram of filtering effect: (**a**) density field $\tilde{\rho }$ before filtering, (**b**) density field ψ after filtering.

In the case of two feed directions as shown in Figure 3, the filtered density field $\psi_{m}$ in each direction should merge so only the intersection area remains. Hence, Equation (15) is adopted.

(15) $$\psi ={\left(\frac{1}{M}\overset{M}{\underset{m=1}{\sum }}{\left(\psi_{m}\right)}^{q}\right)}^{\frac{1}{q}}m=1,2\ldots M$$

In Equation (15), m represents the number of feed directions, and M is the total number of directions. When q→−∞, Equation (15) takes the minimum value of each filtered density field. To ensure the function smoothness, q is set to −4.

By solving Equations (11)–(15), the filtered field is shown in Figure 3b. The void area inside the initial density field is eliminated and all boundary areas can be accessed by the light through the feed directions.

Since $\tilde{\rho }$ is still the physical density field, we set the following constraint to eliminate the gap between $\tilde{\rho }$ and ψ, therefore enabling the directional material change effect.

(16) $$I=\overset{N}{\underset{e=1}{\sum }}\frac{H\left[\psi \left({\tilde{\rho }}_{e}\right)-{\tilde{\rho }}_{e}\right]}{N}\leq 10^{-b}$$

where I represents the unreachable domain by light, N is the total number of elements, and b is a positive number. H is the Heaviside projection function. In order to improve the convergence, Equation (16) can be further developed into the logarithmic form,

(17) $$I=\log_{10}\left(\overset{N}{\underset{e=1}{\sum }}\frac{H\left[\psi \left({\tilde{\rho }}_{e}\right)-{\tilde{\rho }}_{e}\right]}{N}\right)+b\leq 0$$

The first order derivative of I with respect to the design variable $\tilde{\rho }$ can be developed as,

(18) $$\frac{\partial I}{\partial \tilde{\rho }}=\frac{\frac{\partial H\left(\psi -\tilde{\rho }\right)}{\partial \tilde{\rho }}}{\ln\left(10\right)\cdot N\cdot \sum_{e=1}^{N}\frac{H\left[\psi \left({\tilde{\rho }}_{e}\right)-{\tilde{\rho }}_{e}\right]}{N}}$$

where $\frac{\partial H\left(\psi -\tilde{\rho }\right)}{\partial \rho }$ can be expressed by the chain rule as,

(19) $$\frac{\partial H\left(\psi -\tilde{\rho }\right)}{\partial \rho }=\frac{\partial H\left(\psi -\tilde{\rho }\right)}{\partial \left(\psi -\tilde{\rho }\right)}\frac{\partial \left(\psi -\tilde{\rho }\right)}{\partial \tilde{\rho }}=\frac{\partial H\left(\psi -\tilde{\rho }\right)}{\partial \left(\psi -\tilde{\rho }\right)}\left(\frac{\partial \psi }{\partial \tilde{\rho }}-\frac{\partial \tilde{\rho }}{\partial \tilde{\rho }}\right)$$

In Equation (19), $\frac{\partial \psi }{\partial \tilde{\rho }}$ can be obtained by differentiating Equation (15); see Equation (20).

(20) $$\frac{\partial \psi }{\partial \tilde{\rho }}=\overset{M}{\underset{m=1}{\sum }}\left[{\left(\frac{1}{M}\overset{M}{\underset{m=1}{\sum }}{\left(\psi_{m}\right)}^{q}\right)}^{\frac{1}{q}-1}\frac{1}{M}{\left(\psi_{m}\right)}^{q-1}\frac{\partial \psi_{m}}{\partial \tilde{\rho }}\right]$$

And then, $\frac{\partial \psi_{m}}{\partial \tilde{\rho }}$ can be obtained from Equation (11); see Equation (21).

(21) $$\frac{\partial \psi_{m}}{\partial {\tilde{\rho }}_{\;}}=\beta T^{T}\left[K_{S}^{-1}\left(T-\frac{\partial K_{S}}{\partial \tilde{\rho }}K_{S}^{-1}T\tilde{\rho }\right)\right]$$

where $K_{S}$ is the FE stiffness matrix related to solving Equation (11). Finally, the MMA (method of moving asymptotes) [41] optimizer is employed for the design update.

## 3. Numerical Implementation

**Figure 4:**
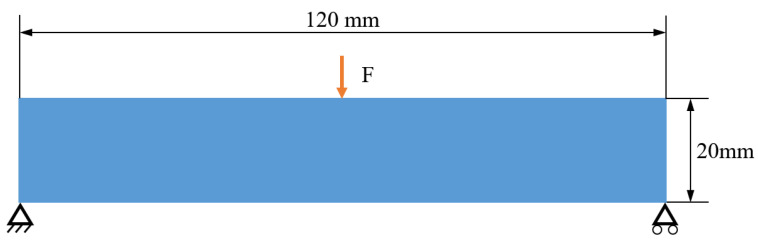
Design domain and boundary conditions of 2D MBB beam.

First, the 2.5D optimization of the benchmark Messerschmitt–Bölkow–Blohm (MBB) beam is conducted. The design domain and boundary conditions are shown in Figure 4. A load of 10 KN is applied at the center of the top edge, and the two bottom corners are supported to limit the horizontal sliding. The design domain has the size of 120 mm × 20 mm and is discretized into 768 × 128 square elements for meshing. The base material parameters include Young’s modulus $E_{0}$ = 1 GPa and Poisson’s ratio μ = 0.3. In the optimization setup, the maximum volume fraction is 0.3. The radius r of the smoothing filter is set to 6 times of the grid size. The β in Heaviside projection increases along the iterations, staring from 1, doubling for every 40 iterations or when the objective function changes less than 0.1%, and ending at the threshold 512. The optimization terminates when the objective function varies less than 0.1% in the consecutive iterations.

**Figure 5:**
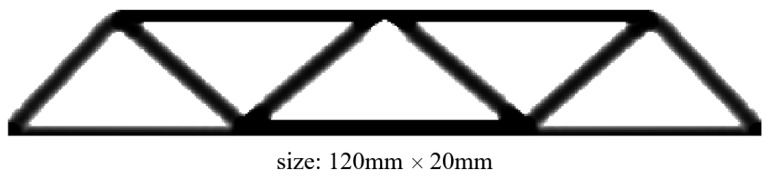
Material distribution of optimized 2D structure.

**Figure 6:**
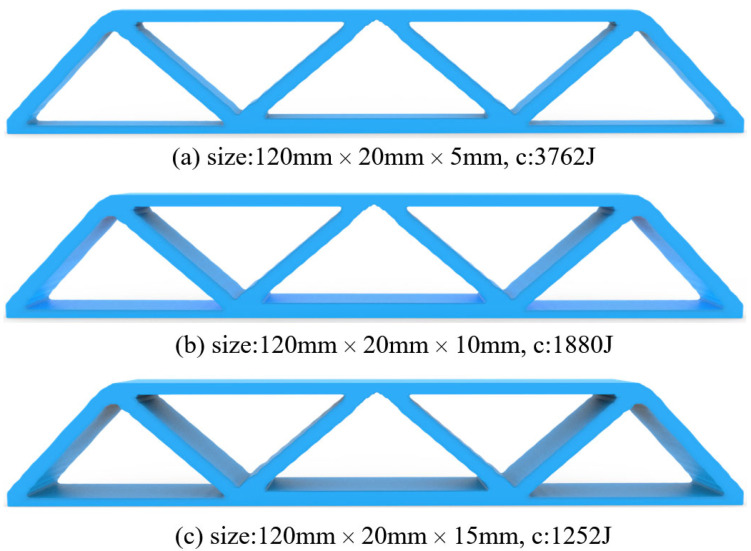
2.5D structures for MBB beam with different thickness, (**a**) Th = 5 mm, (**b**) Th = 10 mm, and (**c**) Th = 15 mm.

Figure 5 gives the optimized material distribution. Then, extruding the 2D structure with thicknesses of 5 mm, 10 mm, and 15 mm derives the 2.5D structures shown in Figure 6, where c represents the strain energy of the structure whose unit is J.

**Figure 7:**
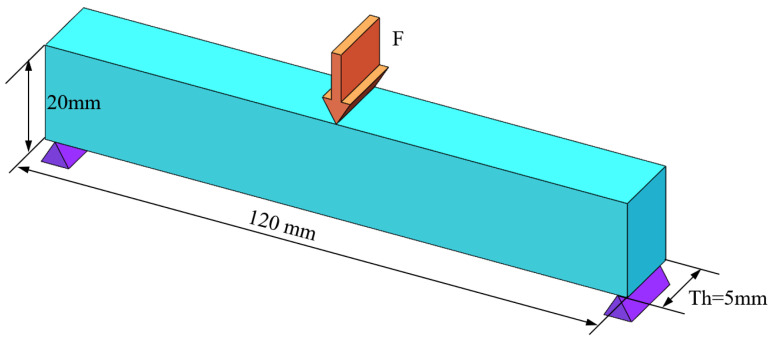
Design domain and boundary conditions of the 3D MBB beam.

**Figure 8:**
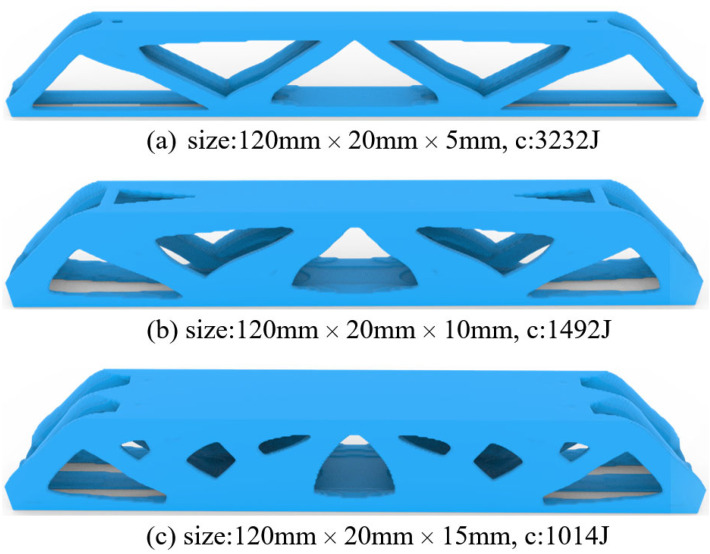
Optimization results subject to different thicknesses of (**a**) Th = 5 mm, (**b**) Th = 10 mm, and (**c**) Th = 15 mm.

**Figure 9:**
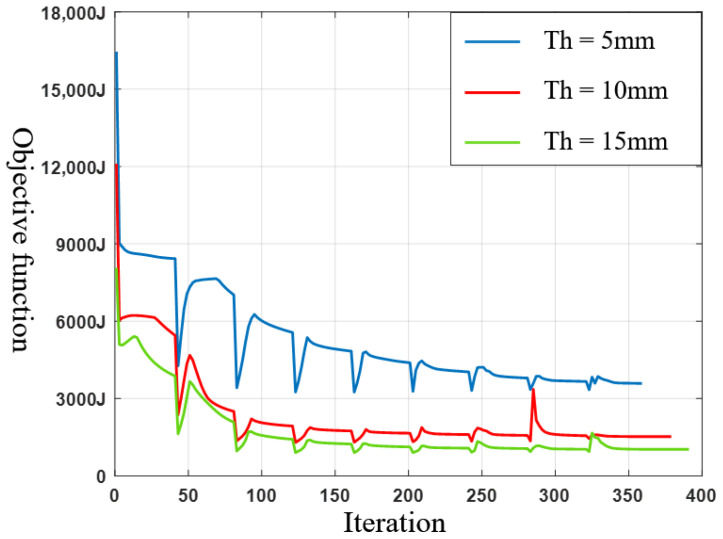
Convergence histories.

Then, the MBB beam is optimized in three-dimensional working conditions, and the design domain and boundary conditions are shown in Figure 7. A total force F = 10 KN is uniformly loaded along the centerline of the top surface. The design domain size is 120 mm × 20 mm × Th, where Th represents thickness of the beam, which is set to 5 mm, 10 mm and 15 mm, respectively. The element size, material properties, maximum volume fraction, filter radius, convergence conditions and other settings are kept the same as the two-dimensional case. Figure 8 shows the optimization results under three thicknesses, and the convergence histories are shown in Figure 9.

**Figure 10:**
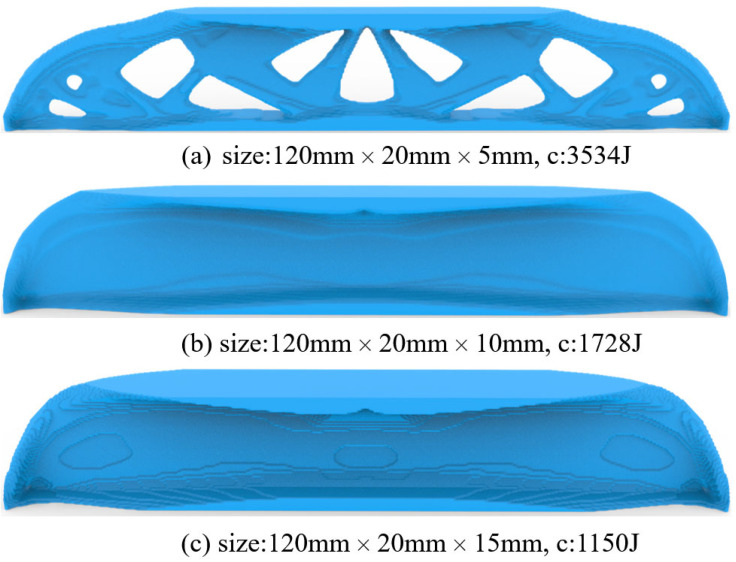
The 3D optimization results with directional material removal subject part thicknesses of (**a**) Th = 5 mm, (**b**) Th = 10 mm, and (**c**) Th = 15 mm.

Finally, the complexity-restricted topological design is derived by forcing directional material adding/removal. Since the part would be laid down and have its thickness direction as the printing direction, we pick up the front-back and back-front orientations for the bi-directional material changes, so that large-area cross-sections would be produced during slicing. Then, the optimized results are shown in Figure 10. It can be seen that, compared to the results in Figure 8, the directional material removal reduces the structural complexity by distributing the majority of materials around the medium surface, from which the predicted structural performances are compromised.

## 4. Material and Experiment

### 4.1. Material

Three groups of optimized models obtained in the above are fabricated by fiber reinforced composite 3D printing. The printing material is carbon fiber reinforced polylactic acid (CFRPLA) from ESUN in Shenzhen, China, which has a filament diameter of 1.75 mm.

### 4.2. Experiment

**Figure 11:**
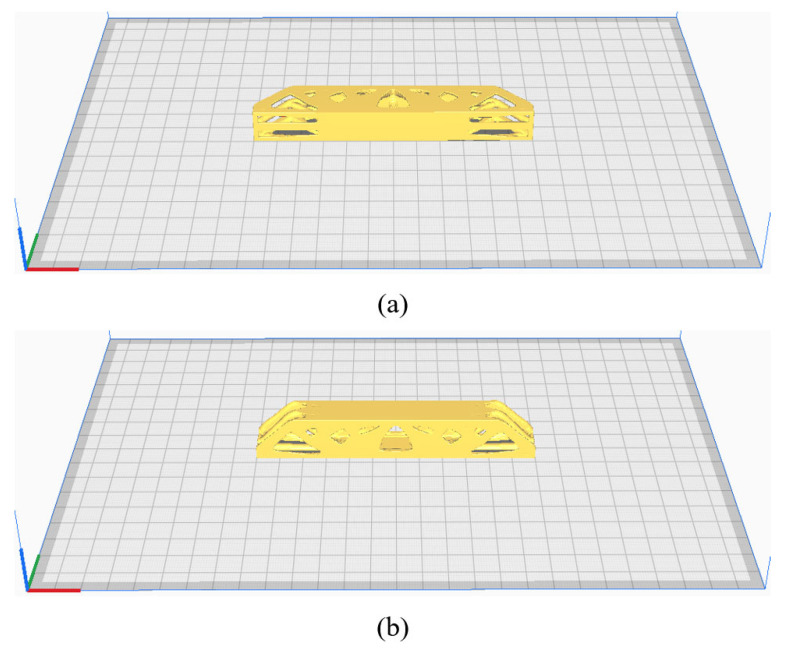
Print direction of the part (**a**) along the thickness direction, (**b**) along the height direction.

The optimized models have a total of three sizes, which are 120 mm × 20 mm × 5 mm, 120 mm × 20 mm × 10 mm and 120 mm × 20 mm × 15 mm, respectively. The JG-E6 FDM printer made by JGMAKER in Shenzhen, China is used, and its nozzle diameter is 0.4 mm. During the printing, the layer thickness is set to 0.2 mm and the printing speed is set to 60 mm/s. The temperatures of printing bed and printing head are maintained at 60 ℃ and 240 ℃, respectively. To further discuss the influence of printing directions, the 3D freeform structures are fabricated through both the thickness and height directions as shown in Figure 11. All the printing paths of specimens are generated using Ultimaker Cura 5.0, that is, each layer of printing paths is composed of two paralleled contours and infilled with straight lines that are parallel to the longitudinal direction of the MBB beam. The hatch space is 0.4 mm.

**Figure 12:**
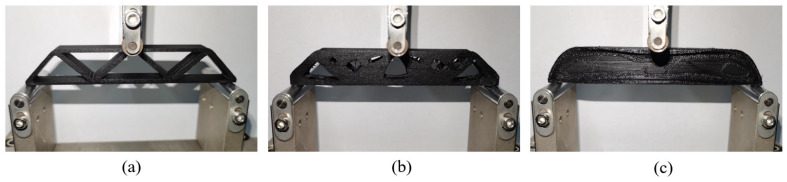
Three-point bending test of different MBB beams: (**a**) 2.5D structure, (**b**) the freeform 3D structure, and (**c**) the simplified 3D structure.

After that, three-point bending tests (see Figure 12) are performed to evaluate the mechanical properties of specimens, according to the test standard ASTM D7249/D7249M-20-1 [42]. The bending tests are conducted using the WDW-20M universal testing machine produced by ZHONGLUCHANG in Jinan, China with a maximum 20 kN load. The length of span is 120 mm, and the loading rate is set to 0.5 mm/min.

## 5. Results and Discussions

**Figure 13:**
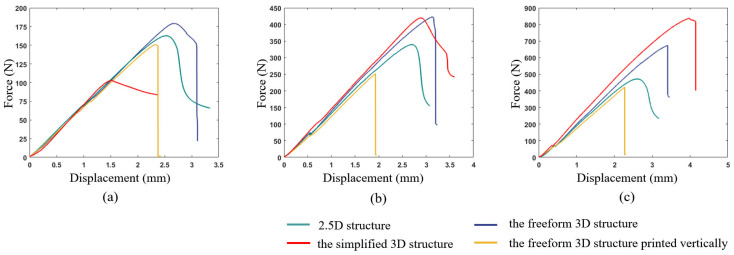
Force–displacement curves for samples with different thicknesses: (**a**) Th = 5 mm, (**b**) Th = 10 mm, and (**c**) Th = 15 mm.

After weighing the printed samples, the results are shown in Table 1. The weight of the samples with the same thickness are basically the same, because the volume fraction is uniformly set to 0.3 in the optimization process. The force–displacement curves of the three-point bending tests are shown in Figure 13, from which the stiffness and strength of the specimens can be compared.

Note in this section, the structure extruded from 2D topology optimization is named the ‘2.5D structure’, the structure derived from 3D topology optimization without complexity constraint is called the ‘freeform 3D structure’, and the structure derived from 3D topology optimization with directional material removal is called the ‘simplified 3D structure’ because of the reduced complexity.

### 5.1. Comparison between 3D and 2.5D Optimized Structures

The 2.5D structures are generated by extruding the 2D optimized pattern with a given thickness. The main advantage they have is that the printing path of each layer is exactly the same, and the interlayer bonding is firm. However, the extrusion operation from the 2D pattern cannot guarantee the optimal solution in 3D, because 3D freeform topology optimization has a larger design space than 2D optimization plus extrusion, that in theory, performs better. By analyzing the green and blue curves in Figure 13, for specimens with the same thickness and printing directions, the load-bearing capacities of the 3D freeform structures are higher than those of the 2.5D structures, which is consistent with the simulation prediction. And the performance differences between 3D freeform structures and 2.5D structures become more evident with the increase in structure thickness, since the design space increases along with the thickening.

**Figure 14:**
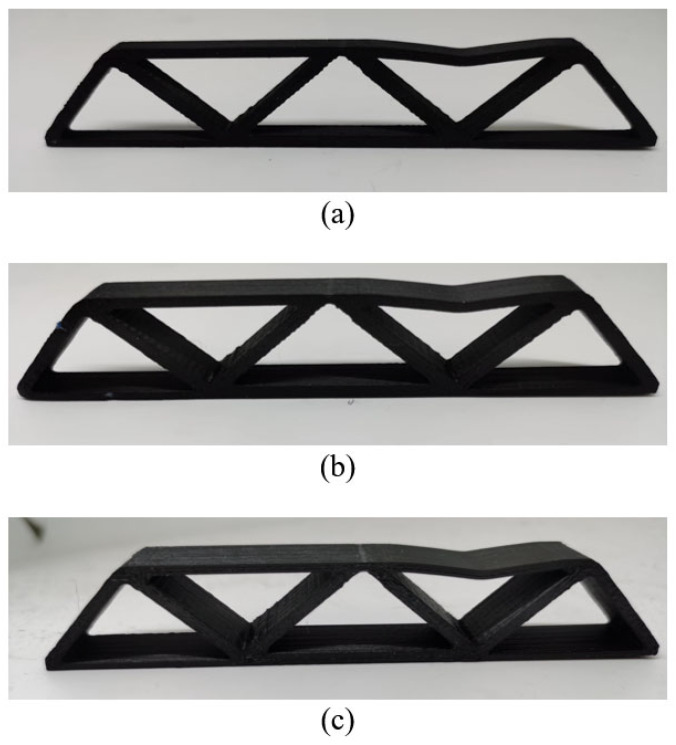
Failures of 2.5D structures with different thicknesses: (**a**) Th = 5 mm, (**b**) Th = 10 mm, and (**c**) Th = 15 mm.

**Figure 15:**
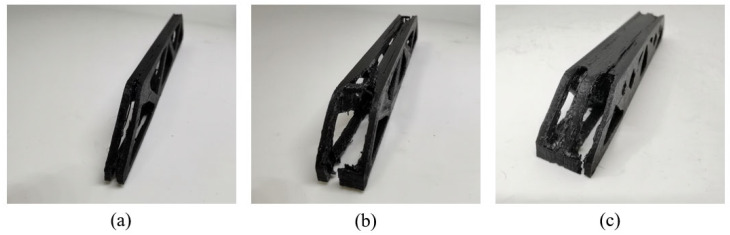
Failures of the freeform 3D structures with different thicknesses: (**a**) Th = 5 mm, (**b**) Th = 10 mm, and (**c**) Th = 15 mm.

Moreover, as shown in Figure 14, the failure form of 2.5D structures with different thicknesses is consistently the buckling of the top surface, while no interlayer fracture happens. On the other hand, the 3D freeform structures fail due to the interlayer fracture (see Figure 15), indicating that the 3D freeform structures have insufficient bonding strength due to reduced cross-section area caused by drastic geometric variations along the thickness direction.

### 5.2. The Influence of Geometric Simplification

**Figure 16:**
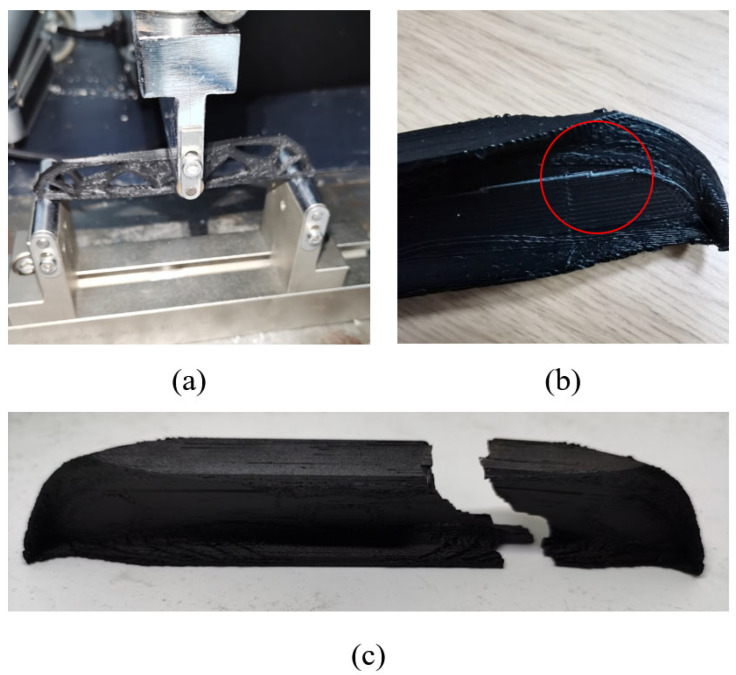
Failures of the simplified 3D structures with different thicknesses: (**a**) Th = 5 mm, (**b**) Th = 10 mm (The failure site marked by the red circle), and (**c**) Th = 15 mm.

Comparing the freeform 3D structure and the simplified 3D structure, the geometrically simplified one has evidently enhanced stiffness and strength performance at the thickness of 15 mm, which is contrary to the simulation prediction. Referring to Figure 15c, the freeform 3D structure fails due to the interlayer fracture, indicating insufficient bonding strength due to reduced cross-section area. However, the simplified 3D structure has increased cross-section area due to the concentration of materials at the symmetry plane, which enhances the interlayer bonding, as shown in Figure 16c. At the thickness of 10 mm, the simplified 3D structure still shows better stiffness, but the strength falls close to that of the freeform 3D structure. The reason lies in local buckling due to the severely reduced plate thickness (see Figure 16b). The simplified 3D structure of thickness 5 mm has obviously degraded strength because of the out-of-plane buckling at the early stage loading (see Figure 16a).

### 5.3. The Influence of Printing Direction

**Figure 17:**
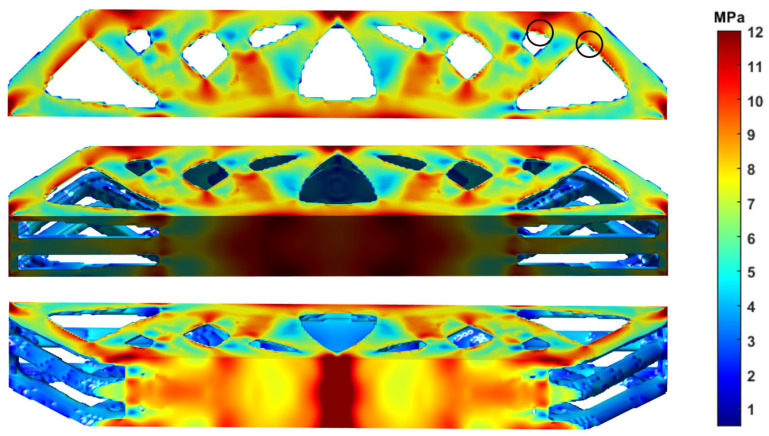
The von Mises stress distribution of the 3D freeform structure with thickness of 15 mm calculated by finite element analysis.

**Figure 18:**
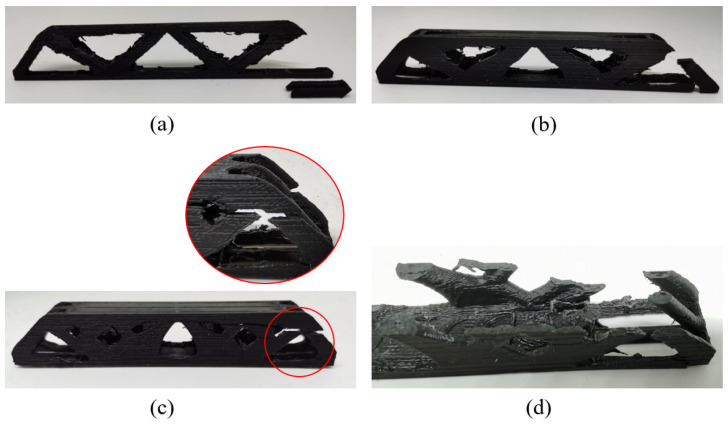
Failures of the freeform 3D structures printed vertically with different thicknesses: (**a**) Th = 5 mm, (**b**) Th = 10 mm, (**c**) Th = 15 mm, and (**d**) inter-layer interface of failure area with Th = 15 mm.

The printing direction has a great impact on the mechanical performance. As shown in Figure 13, 3D structures printed vertically have poor strength performances that even fall below the 2.5D structures. This conclusion is consistent in all three groups of experiments. Figure 17 shows the von-Mises stress distribution of the 3D freeform structure with thickness of 15 mm, and the highly stressed areas are marked in dark red color. Due to the well-known staircase effect, if printed vertically, the staggered structural boundaries inside the black circles of Figure 17 amplify the local stress concentration that initializes the cracks. Then, the cracks expand quickly along the interlayer interfaces due to the insufficient bonding strength, forming the horizontal fractures as depicted in Figure 18c. As shown in Figure 18d, the horizontal cross-section is composed of a few isolated islands with small adhesion areas, which are prone to manufacturing defects and they perform poorly in bearing loads. The same explanations apply to other vertically printed freeform 3D structures even though the thickness varies, and consequently, the bending strength degradation due to vertical printing is explained.

### 5.4. Discussion

In summary, regarding topology optimization for 3D-printed fiber composites, the structure with great complexity is not suggested since it is very likely that isolated small islands would appear at slicing, which are prone to printing defects and unqualified interlayer bonding. Hence, the actual mechanical performance would be compromised than the theoretical prediction. Restricting the structural complexity has been proven an effective manner of strengthening the design; however, the exact directions for material removal would be case-based and also tightly bonded to the printing direction, for which a general algorithm of determining these directions should be further disclosed.

## 6. Conclusions

This work investigates the physical mechanical properties of a group of 3D-printed fiber reinforced composite structures with different levels of geometric complexity. Three types of topology optimization algorithms, 2.5D topology optimization, 3D topology optimization, and 3D topology optimization with directional material removal, are used to generate test samples. Through the three-point bending test, the stiffness, strength and failure of all samples are obtained. It can be concluded from the experiments that (1) the bearing capacity of the three-dimensional structure is higher than that of the 2.5-dimensional structure. (2) The stiffness and strength performance of the geometrically simplified 3D structure are obviously improved when the thickness is large, while when the thickness is small, the strength will be weakened due to buckling. (3) After changing the printing direction, the strength performance of the freeform 3D structure is poor, even lower than the 2.5D structure. It is clear from the experimental results that the printing direction and manufacturing quality has a great influence on the actual mechanical performance of 3D-printed fiber composites. Although structures with high geometric complexity have a better theoretical performance, the detailed structures are difficult to fabricate perfectly by current manufacturing technology. In this example, the printing defects and unqualified interlayer bonding caused by FDM additive manufacturing impede the realization of the optimally predicted mechanical performance. Restricting the structural complexity is helpful to alleviate the printing defects by FDM additive manufacturing. Therefore, the manufacturing constraints and defects should be considered in the design and optimization procedure to enhance the actual performance of 3D-printed fiber composite parts.

## Figures and Tables

**Table 1:** Printed samples and their weights.

Samples	Thickness (mm)	Weight (g)
2.5D structures	5	4.42
	10	8.88
	15	13.32
The freeform 3D structures	5	4.51
	10	9.12
	15	13.94
The simplified 3D structures	5	4.45
	10	8.95
	15	12.95
The freeform 3D structures printed vertically	5	4.88
	10	9.45
	15	14.2

## Data Availability

Data will be made available on request.
